# DNA Double Strand Breaks and Chromosomal Translocations Induced by DNA Topoisomerase II

**DOI:** 10.3389/fmolb.2019.00141

**Published:** 2019-12-10

**Authors:** Fernando Gómez-Herreros

**Affiliations:** ^1^Instituto de Biomedicina de Sevilla (IBiS), Hospital Virgen del Rocío-CSIC-Universidad de Sevilla, Seville, Spain; ^2^Departamento de Genética, Universidad de Sevilla, Seville, Spain

**Keywords:** DSB repair, DNA topoisomerase II, chromosomal translocations, genome instability, transcription

## Abstract

DNA double strand breaks (DSBs) are the most cytotoxic lesions of those occurring in the DNA and can lead to cell death or result in genome mutagenesis and chromosomal translocations. Although most of these rearrangements have detrimental effects for cellular survival, single events can provide clonal advantage and result in abnormal cellular proliferation and cancer. The origin and the environment of the DNA break or the repair pathway are key factors that influence the frequency at which these events appear. However, the molecular mechanisms that underlie the formation of chromosomal translocations remain unclear. DNA topoisomerases are essential enzymes present in all cellular organisms with critical roles in DNA metabolism and that have been linked to the formation of deleterious DSBs for a long time. DSBs induced by the abortive activity of DNA topoisomerase II (TOP2) are “trending topic” because of their possible role in genome instability and oncogenesis. Furthermore, transcription associated TOP2 activity appears to be one of the most determining causes behind the formation of chromosomal translocations. In this review, the origin of recombinogenic TOP2 breaks and the determinants behind their tendency to translocate will be summarized.

## Introduction

Chromosomal translocations are rearrangements of large fragments of DNA. When transcribed regions are affected, genome translocations usually result in the inactivation of one or a group of genes with the consequent deleterious effects for cellular survival. However, on occasion, translocations can generate chimeric proteins or deregulate transcription programmes creating abnormal growth capacities and contributing to malignancy and tumor development. Over 10,000 gene fusions have been found in cancer, most of which are considered passenger mutations, consequence of the intrinsic instability of tumor progression. Among them, more than 300 are recurrent and contribute to initial stages of cellular disarray (Mitelman et al., 2007; Mertens et al., 2015).

Recurrence of chromosomal translocations is determined by a large number of factors, starting from the nature of the DNA break and including the pathway involved in its repair, the cell cycle stage, the chromatin status of the locus, and the genomic location of the lesion. Since most of these factors are dynamic and interconnected, their relative relevance is difficult to establish, and many aspects of the origin of genomic translocations remain unclear. Recent studies have unveiled that transcription and 3D organization of the genome are two major determinants in the appearance of DNA double-stranded breaks (DSBs) and they promote chromosomal translocations. In this brief review how DNA topoisomerase II (TOP2) appears at the crossroad of these factors will be discussed.

## DNA Transactions and TOP2 Activity

DNA topoisomerases are essential enzymes present in all cellular organisms with critical roles in DNA metabolism. DNA topoisomerases release the torsional stress generated in the DNA by a wide variety of processes including replication, transcription, 3D genome organization, and chromosome segregation (Pommier et al., 2016). According to their mechanism of action, DNA topoisomerases are classified in two types depending on whether they cut one (type I) or two strands (type II) of the DNA double helix. TOP2 is a type II enzyme that can pass an intact DNA duplex through a broken one while covalently bound to the DNA. Once strand passage is completed, the enzyme reseals the break (Nitiss, 2009a). Vertebrates express two TOP2 isoforms, TOP2α and TOP2ß. While TOP2ß is expressed throughout the cell cycle, TOP2α levels correlate with cellular proliferation and peak at S and G2/M phases. TOP2α has a major role in replication and chromosome segregation. TOP2ß activity has been mainly associated to transcription. It participates in: transcription elongation, conserving the structure of either active or inactive promoters, promoting the activation of hormone-driven, and early response genes and in the release of paused RNA polymerases (Ju, 2006; Haffner et al., 2010; Madabhushi et al., 2015; Dellino et al., 2019).

**Figure 1:**
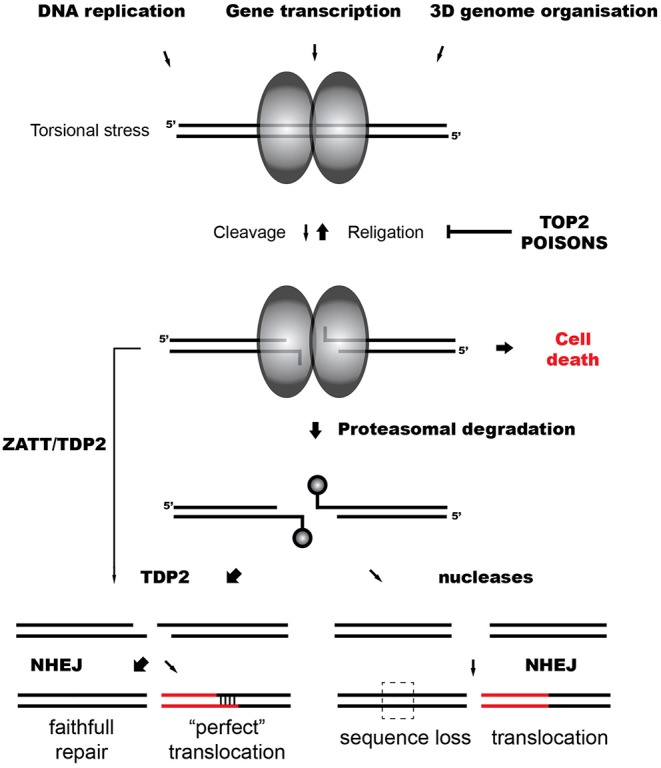
TOP2-induced chromosomal translocations. Model representing the repair of TOP2 abortive breaks and the influence of TDP2-dependent and independent NHEJ on TOP2-induced translocations.

A key intermediate of topoisomerase activity is the cleavage complex (TOP2cc), formed when the topoisomerase cleaves the DNA and each subunit of the TOP2 dimer becomes covalently linked to the 5′-terminus of the break via a phosphotyrosyl bond (Vos et al., 2011) (Figure 1). Although the cleavage complex is normally transient, naturally due to unclear circumstances or induced by the presence of anti-tumor agents that act as topoisomerase “poisons” the cleaved intermediate can result in the formation of abortive (irreversible) TOP2cc, a singular DSB (Deweese and Osheroff, 2009; Nitiss, 2009b).

## TOP2 and Oncogenic Translocations

TOP2-associated translocations are main drivers of some common hematological and solid tumors (Felix et al., 2006; Haffner et al., 2010). Oncogenic translocations related to TOP2 have been mainly associated to TOP2ß activity (Nitiss, 2009a; Pommier et al., 2016; Madabhushi, 2018). However, after many years of study, we only start to understand the molecular mechanisms that direct TOP2-induced rearrangements.

In prostate cancer, androgen-regulated genes are frequently fused to transcription factors of the ETS family. For instance, the fusion of *TMPRSS2* and *ERG* occurs in more than 50% of prostate malignancies resulting in a hormone-dependent expression of *ERG* in prostate tissue (Kumar-Sinha et al., 2008). *TMPRSS2* and *ERG* expression has been linked to TOP2 activity since TOP2ß participates in the androgen-dependent activation of these genes. Androgen signaling promotes co-recruitment of androgen receptor and TOP2ß to *TMPRSS2* and *ERG* breakpoints, which can trigger recombinogenic DSBs (Haffner et al., 2010).

Recurrent fusions involving *MLL* and members of the super-elongation complex, such as *AF4* and *AF9*, account for 10–30% of secondary and infant acute myeloid leukemia (AML) (Mitelman et al., 2007; Mertens et al., 2015). Numerous potential mechanisms for *MLL* breakage have been proposed, from Alu-mediated recombination to TOP2ß-induced breaks (Cowell and Austin, 2012; Wright and Vaughan, 2014). Notably, secondary leukemias are those resulting from the use of genotoxic chemotherapeutical drugs, mainly alkylating agents or TOP2 inhibitors, uplifting the direct connection between TOP2 and translocations in *MLL* (Wright and Vaughan, 2014). The link of infant leukemia with TOP2 abortive activity is less clear but a correlation with dietary flavonoids, natural TOP2 poisons, has been proposed (Ross, 2000).

## The Contribution of TOP2 in the Cellular Pool of DSBs

The first factor influencing the propensity of a region to translocate is the frequency of DNA breakage. DSBs can arise directly from exogenous threats (clastogens), such as radiation and chemotherapeutic or industrial chemicals. Endogenous threats are stochastic activity of apoptotic caspases, nucleases such as RAG1 and RAG2, and TOP2 (Ashour et al., 2015; Lieber, 2016). DSBs can also form indirectly from coincident single strand breaks (SSBs), induced exogenously by alkylating chemotherapeutical agents, or naturally by type I DNA topoisomerases, reactive oxygen species (ROS), or activation-induced cytidine deaminase (AID) (Xu et al., 2012; Rulten and Caldecott, 2013). DNA replication across SSBs also generates single ended DSBs (Kuzminov, 2001). This is a prominent source of DSBs, since SSBs are known to be as frequent as 50,000 per day per cell (Lindahl, 1993). Finally, replication stress, due to replication fork encountering with inter-strand crosslinks or non-B forms of DNA such as RNA-DNA hybrids (R-loops), is also known to promote DSB formation (Gómez-González and Aguilera, 2019).

The most precise information about endogenous DSBs comes from non-biased DSB mapping methods, developed to evaluate illegitimate cleavage by RAG nucleases, and AID in B cells or by CRISPR-Cas (Chiarle et al., 2011; Crosetto et al., 2013; Tsai et al., 2014; Frock et al., 2015; Canela et al., 2016; Lensing et al., 2016; Yan et al., 2017). These studies draw two major conclusions. The first one is that recurrent translocations (typically those that drive specific cancers) are mostly tissue-specific and triggered by recurrent DSBs. For instance, RAG off-target sites have been efficiently detected in activated mouse B-cells in which Rag1 and Rag2 are induced, supporting the role of stochastic activity of these nucleases in the formation of B-cell specific DSBs (Kuo and Schlissel, 2009; Chiarle et al., 2011; Canela et al., 2016).

The second conclusion of these studies, and probably the most ground-breaking, is that there are more stochastic sources of DSBs that are not cell-cycle nor tissue-specific but can be consistently detected in mice and human cells. Some of these are related to replication stress and frequently appear in long gene bodies, which are prone to undergo late replication and are predisposed to replication-transcription conflicts (Canela et al., 2016; Wei et al., 2016). Notably, others, a bulky group of them (over 60%), increase in frequency in the presence of the TOP2 poison etoposide (Canela et al., 2017). These breakpoints, concentrate in chromatin loop boundaries, gene bodies and promoter-proximal locations, frequently transcription start sites (TSS) (Chiarle et al., 2011; Schwer et al., 2016; Yan et al., 2017).

But, what is the origin of these DSBs? TOP2ß is positioned at loop anchors, this is, CTCF/cohesin (RAD21) binding sites that flank topologically associating domains, suggesting that it might be required to solve topological problems during loop extrusion dynamics (Uusküla-Reimand et al., 2016). Etoposide-induced TOP2cc can be detected in these loci, independently of transcription and replication activities (Canela et al., 2017). But these are reversible TOP2cc. Contrary, detection of abortive TOP2cc (irreversible) by DSB or protein-linked mapping has demonstrated that the induction of DSBs at loop anchors by TOP2 activity is largely depend on active transcription (Canela et al., 2019; Gittens et al., 2019; Gothe et al., 2019). In fact, a large number of TOP2ß-associated breaks also concentrate in gene bodies and around TSS, independently of RAD21 (Chiarle et al., 2011; Schwer et al., 2016; Canela et al., 2017; Yan et al., 2017; Gittens et al., 2019; Gothe et al., 2019). Importantly, distribution of TOP2 breaks around TSS positively correlates with transcription levels at these loci (Gittens et al., 2019). Indeed, the inhibition of transcription elongation prevents TOP2 breakage at these loci suggesting that transcription is a major driving force in TOP2 abortive cycles (Gómez-Herreros et al., 2017; Gothe et al., 2019). For instance, breaks at TSS associate with promoter fragility suggesting that events such as RNA polymerase II pause release requires TOP2 activity and is a source of DSBs (Dellino et al., 2019).

Regarding TOP2 isoforms, both TOP2α and TOP2ß influence DNA breakage at these hotspots (Yu et al., 2017; Gothe et al., 2019). Intriguingly, and despite a similar localization of both isoforms, TOP2ß-lacking cells reduce breakage at these loci, suggesting a dominant role of TOP2ß over TOP2α (Cowell et al., 2012; Canela et al., 2017).

In theory, any DSB can be a potential origin of a rearrangement. Interestingly, oncogenic breakpoints such as those found in *TMPRSS2, ERG, MLL, AF4*, and *AF9*, among many others, are localized to TOP2ß/CTCF/RAD21 breakpoints (Canela et al., 2017, 2019; Gothe et al., 2019). Moreover, TOP2-induced breaks have been detected by high-throughput, genome-wide translocation sequencing (HTGTS). HTGTS “fish” breaks genome wide using a bait DSB in a controlled locus (Chiarle et al., 2011; Frock et al., 2015). HTGTS has revealed the tendency of TOP2-induced breaks to translocate, with highly transcribed genes translocating more than with non-transcribed ones (Chiarle et al., 2011; Wei et al., 2016; Canela et al., 2019). Some of these hotspots are localized in TSS (Schwer et al., 2016).

## Illegitimate TOP2 DSB Repair

The illegitimate repair of DNA ends *in trans* is mediated by DNA repair pathways, but how often and why are breaks incorrectly joined is not clear. The two major pathways involved in the repair of DSBs in eukaryotic cells are non-homologous end joining (NHEJ) and homologous recombination (HR). HR occurs specifically in late S and G2 phases of the cell cycle, as it requires the presence of a sister chromatid for the repair process (Mehta and Haber, 2014; Wright et al., 2018). HR is considered an error-free pathway due to the fact that a very large homology, up to megabases, is used, ensuring the accuracy of the repair (Symington, 2016). The occurrence of recombination between homologous chromosomes or tandem repeats has been shown to be substantially low and HR-deficient cells exhibit higher rates of genome rearrangements, historically absolving HR for almost any responsibility in chromosomal translocations (Moynahan and Jasin, 1997, 2010; Lambert et al., 1999; Stark and Jasin, 2003). Exceptions to the HR paradigm are Rad51-independent but homology-directed pathways, Break-Induced Replication (BIR) and single strand annealing (SSA), which can promote exchanges *in trans* (Elliott et al., 2005; Malkova and Ira, 2013; Bhargava et al., 2016; Sakofsky and Malkova, 2017; Kramara et al., 2018).

In contrast to HR, NHEJ is active throughout the cell cycle and involves the efficient ligation of DNA ends with minimal processing at the site of joining. NHEJ is considered an error-prone pathway since cellular nucleases trim DNA ends to make them compatible before ligation (Lieber, 2010). The homology required in this route is reduced to 1–2 nucleotides, in case there is any, making NHEJ the ideal scapegoat to provoke illegitimate joinings (Chang et al., 2017). However, in the absence of KU70/80 or XRCC4-LIG4, core factors of canonical NHEJ (cNHEJ), a genetically-different, alternative NHEJ (altNHEJ) pathway takes over (Yan et al., 2007). altNHEJ is characterized by a longer homology requirement at the site of break that goes up to 10–20 bp (McVey and Lee, 2008). Resection is mediated by CtIP and the MRN complex, similarly to initial stages of HR (Zhang and Jasin, 2010; Ghezraoui et al., 2014). Base pairing *in trans* of these ends results in translocations characterized by short microhomologies (Guirouilh-Barbat et al., 2004; Kent et al., 2015; Mateos-Gomez et al., 2015; Sfeir and Symington, 2015; Zahn et al., 2015).

In the case of TOP2-induced DSBs, trapped TOP2 represents a particular barrier for ligation, and DNA ends need to be processed. Abortive TOP2cc are denatured and degraded by the proteasome, leaving a TOP2-derived peptide of unknown length covalently bound to the 5′ phosphate of the DNA through a tyrosine residue (Zhang et al., 2006; Lin et al., 2008) (Figure 1). This protein adduct is a hallmark of TOP2 breaks and, like other blocking lesions, can condition DSB repair (Álvarez-Quilón et al., 2014). Resection can generate proficient substrates for HR independently of the nature of the DNA end and potentially remove these adducts. In accordance, HR-deficient cells are hypersensitive to TOP2 poisons, suggesting that TOP2 breaks can be repaired by HR when available (Gómez-Herreros et al., 2013). However, remaining topoisomerase can be precisely removed by Tyrosyl DNA phosphodiesterase 2 (TDP2), which cleaves the phosphotyrosyl bond between the tyrosine and the 5′ phosphate of the DNA (Cortés-Ledesma et al., 2009; Zeng et al., 2010). TDP2 can also remove non-degraded TOP2 in a proteasome-parallel route stimulated by the SUMO-ligase ZATT (Schellenberg et al., 2017). Once TOP2 is removed by TDP2, remaining four base-pair cohesive overhang is ready to be ligated by cNHEJ (Gómez-Herreros et al., 2013) (Figure 1).

TDP2-mediated pathways protect cells from TOP2 abortive activity, accelerating TOP2 DSB repair and preventing cell death and genome instability induced by TOP2 poisons (Gómez-Herreros et al., 2014, 2017; Zagnoli-Vieira et al., 2018). In accordance, breaks in *MLL* induced by the abortive activity of TOP2 during transcription accumulate in cells lacking TDP2 (Gómez-Herreros et al., 2017). Notably, TDP2 facilitates a faithful repair of TOP2 breaks suppressing chromosomal translocations generated by TOP2 during transcription (Gómez-Herreros et al., 2017) (Figure 1). Intriguingly, TDP2-processed ends can also originate chromosomal translocations. A four base pair homology at break joining can be generated in cells treated with etoposide and is dependent on TDP2 (Gómez-Herreros et al., 2017) (Figure 1). About 20% of secondary AML is characterized by this type of junction that is referred to as “perfect” (Whitmarsh et al., 2003; Meyer et al., 2005, 2017). A very high number of DSBs might challenge physiological repair capacities and promote this illegitimate pairing.

Since TDP2 prevents genome instability and chromosomal translocations, it has been hypothesized that marginal routes would promote them (Caldecott, 2012; Gómez-Herreros et al., 2013, 2017). If HR is not available, in contrast to the “clean” end processing mediated by TDP2, endonucleases would potentially generate the loss of information at DNA ends (Figure 1). However, it has been shown that MRE11, the nuclease activity of the MRN complex, can process abortive TOP2 DSBs regulated by a HR-independent role of BRCA1 (Hoa et al., 2016; Sasanuma et al., 2018). Notably, MRE11 H129N (nuclease deficient) mutants exhibit increased instability and translocations when treated with TOP2 poisons (Sasanuma et al., 2018; Gothe et al., 2019). The contribution of MRE11 and other nucleases such as ARTEMIS in the repair of physiological levels of TOP2 breaks, their relevance in TOP2 poison-based chemotherapy and their implication in TOP2-induced genome instability is under discussion.

Contrary to mouse cells, in which translocations depend mostly on altNHEJ, cNHEJ mediates translocations induced by nucleases and ionizing irradiation in humans (Ghezraoui et al., 2014; Biehs et al., 2017; So and Martin, 2019). However, it has also been shown that the DSB structure can predispose repair toward cNHEJ and altNHEJ suggesting that the nature of the DNA end can condition its repair (So and Martin, 2019). The role of NHEJ in the formation of TOP2-induced translocations is controversial. An epistatic effect of Ku70 over TDP2 in etoposide sensitivity in avian cells suggests that cNHEJ mediates TOP2-induced DSB repair (Gómez-Herreros et al., 2013). However LIG4 deficiency increases *MLL* translocations suggesting that different pathways such as altNHEJ might mediate TOP2 induced rearrangements in the absence of cNHEJ (Gothe et al., 2019). Further research is required to clarify this point.

Noteworthily, despite in the presence of a sister chromatid NHEJ still has a dominant role (Beucher et al., 2009; Karanam et al., 2012), it has been shown that transcriptionally-active regions are preferentially repaired by HR, promoted by open chromatin marks (Aymard et al., 2014; Pfister et al., 2014). This mechanism may moderate mutagenic pathways during the repair of critical sequences. Why TOP2-induced DSBs during transcription are so dependent on TDP2 and NHEJ remains unknown.

## The Synapsis of TOP2 Breaks

A major determinant for translocation propensity is the proximity between donor and acceptor DSBs (Roukos et al., 2013; Hu et al., 2016). Translocations occur preferentially in *cis* and are enhanced within the same topological domain due to pre-existing spatial proximity (Zhang et al., 2012). Notably, transcription may not only mediate TOP2 breakage but break proximity as well. Oncogenic translocation partners are known to share transcription factories (discrete concentrations of actively transcribed genes) (Ghamari et al., 2013). That is the case for *IgH* and *MYC* in Burkitt's lymphoma but also for TOP2 hotspots such as *TMPRSS2* and *ERG* in prostate and *MLL, AF4*, and *AF9* in bone marrow (Osborne et al., 2007; Lin et al., 2009; Cowell et al., 2012). Nevertheless, for other pairs such as *MLL* and *ENL*, an inherent proximity exists, favoring synapsis independently of transcription (Gothe et al., 2019). The association of TOP2 breaks to loop anchors might also contribute to spatial proximity. However, a rational comparison of the 3D architecture of the genome with the genome-wide data of TOP2 abortive breaks is still missing.

Additionally, in G1, repair in highly transcribed loci is delayed and breaks dynamically cluster (Aten et al., 2004). The functional reason for this grouping remains unclear but the absence of a sister chromatid and the preferential use of HR over NHEJ would be an explanation (Aymard et al., 2017). However, grouping unrepaired, and may be partially resected, DSBs seems counter-productive for genome stability. Favoring TDP2-mediated repair in TOP2 breaks associated to transcription may be a mechanism to prevent this situation.

## Conclusion

High transcription and recurrent DSBs are hallmarks of oncogenic hotspots. These two factors get together with spatial proximity and NHEJ-mediated repair in transcription-associated TOP2 breaks generating the perfect breeding ground for chromosomal translocations.

## Author Contributions

FG-H conceived this review and wrote the manuscript.

### Conflict of Interest

The author declares that the research was conducted in the absence of any commercial or financial relationships that could be construed as a potential conflict of interest.

## References

[B1] Álvarez-Quilón A. Serrano-Benítez A. Lieberman J. A. Quintero C. Sánchez-Gutiérrez D. Escudero L. M.. (2014). ATM specifically mediates repair of double-strand breaks with blocked DNA ends. Nat. Commun. 5:3347. 10.1038/ncomms4347 24572510 PMC3948078

[B2] Ashour M. E. Atteya R. El-Khamisy S. F. (2015). Topoisomerase-mediated chromosomal break repair: an emerging player in many games. Nat. Rev. Cancer 15, 137–151. 10.1038/nrc3892 25693836

[B3] Aten J. A. Stap J. Krawczyk P. M. van Oven C. H. Hoebe R. A. Essers J.. (2004). Dynamics of DNA double-strand breaks revealed by clustering of damaged chromosome domains. Science 303, 92–95. 10.1126/science.1088845 14704429

[B4] Aymard F. Aguirrebengoa M. Guillou E. Javierre B. M. Bugler B. Arnould C.. (2017). Genome-wide mapping of long-range contacts unveils clustering of DNA double-strand breaks at damaged active genes. Nat. Struct. Mol. Biol. 24, 353–361. 10.1038/nsmb.3387 28263325 PMC5385132

[B5] Aymard F. Bugler B. Schmidt C. K. Guillou E. Caron P. Briois S.. (2014). Transcriptionally active chromatin recruits homologous recombination at DNA double-strand breaks. Nat. Struct. Mol. Biol. 21, 366–374. 10.1038/nsmb.2796 24658350 PMC4300393

[B6] Beucher A. Birraux J. Tchouandong L. Barton O. Shibata A. Conrad S.. (2009). ATM and Artemis promote homologous recombination of radiation-induced DNA double-strand breaks in G2. EMBO J. 28, 3413–3427. 10.1038/emboj.2009.276 19779458 PMC2752027

[B7] Bhargava R. Onyango D. O. Stark J. M. (2016). Regulation of single-strand annealing and its role in genome maintenance. Trends Genet. 32, 566–575. 10.1016/j.tig.2016.06.007 27450436 PMC4992407

[B8] Biehs R. Steinlage M. Barton O. Juhász S. Künzel J. Spies J.. (2017). DNA double-strand break resection occurs during non-homologous end joining in g1 but is distinct from resection during homologous recombination. Mol. Cell 65, 671–684. 10.1016/j.molcel.2016.12.016 28132842 PMC5316416

[B9] Caldecott K. W. (2012). Tyrosyl DNA phosphodiesterase 2, an enzyme fit for purpose. Nat. Struct. Mol. Biol. 19, 1212–1213. 10.1038/nsmb.2455 23211766

[B10] Canela A. Maman Y. Huang S. Y. N. Wutz G. Tang W. Zagnoli-Vieira G.. (2019). Topoisomerase II-induced chromosome breakage and translocation is determined by chromosome architecture and transcriptional activity. Mol. Cell 75, 252–266. 10.1016/j.molcel.2019.04.030 31202577 PMC8170508

[B11] Canela A. Maman Y. Jung S. Wong N. Callen E. Day A.. (2017). Genome organization drives chromosome fragility. Cell 170, 507–521. 10.1016/j.cell.2017.06.034 28735753 PMC6133249

[B12] Canela A. Sridharan S. Sciascia N. Tubbs A. Meltzer P. Sleckman B. P.. (2016). DNA breaks and end resection measured genome- wide by end sequencing. Mol. Cell 63, 898–911. 10.1016/j.molcel.2016.06.034 27477910 PMC6299834

[B13] Chang H. H. Y. Pannunzio N. R. Adachi N. Lieber M. R. (2017). Non-homologous DNA end joining and alternative pathways to double-strand break repair. Nat. Rev. Mol. Cell Biol. 18, 495–506. 10.1038/nrm.2017.48 28512351 PMC7062608

[B14] Chiarle R. Zhang Y. Frock R. L. Lewis S. M. Molinie B. Ho Y. J.. (2011). Genome-wide translocation sequencing reveals mechanisms of chromosome breaks and rearrangements in B cells. Cell 147, 107–119. 10.1016/j.cell.2011.07.049 21962511 PMC3186939

[B15] Cortés-Ledesma F. El-Khamisy S. F. Zuma M. C. Osborn K. Caldecott K. W. (2009). A human 5'-tyrosyl DNA phosphodiesterase that repairs topoisomerase-mediated DNA damage. Nature 461, 674–678. 10.1038/nature08444 19794497

[B16] Cowell I. G. Austin C. A. (2012). Mechanism of generation of therapy related leukemia in response to anti-topoisomerase II agents. Int. J. Environ. Res. Public Health 9, 2075–2091. 10.3390/ijerph9062075 22829791 PMC3397365

[B17] Cowell I. G. Sondka Z. Smith K. Lee K. C. Manville C. M. Sidorczuk-Lesthuruge M.. (2012). Model for MLL translocations in therapy-related leukemia involving topoisomerase IIβ-mediated DNA strand breaks and gene proximity. Proc. Natl. Acad. Sci. U.S.A. 109, 8989–8994. 10.1073/pnas.1204406109 22615413 PMC3384169

[B18] Crosetto N. Mitra A. Silva M. J. Bienko M. Dojer N. Wang Q.. (2013). Nucleotide-resolution DNA double-strand break mapping by next-generation sequencing. Nat. Methods 10, 361–365. 10.1038/nmeth.2408 23503052 PMC3651036

[B19] Dellino G. I. Palluzzi F. Chiariello A. M. Piccioni R. Bianco S. Furia L. (2019). Release of paused RNA polymerase II at specific loci favors DNA double-strand-break formation and promotes cancer translocations. Nat. Genet. 47, 1–21. 10.1038/s41588-019-0421-z 31110352

[B20] Deweese J. E. Osheroff N. (2009). The DNA cleavage reaction of topoisomerase II: wolf in sheep's clothing. Nucleic Acids Res. 37, 738–748. 10.1093/nar/gkn937 19042970 PMC2647315

[B21] Elliott B. Richardson C. Jasin M. (2005). Chromosomal translocation mechanisms at intronic alu elements in mammalian cells. Mol. Cell 17, 885–894. 10.1016/j.molcel.2005.02.028 15780943

[B22] Felix C. A. Kolaris C. P. Osheroff N. (2006). Topoisomerase II and the etiology of chromosomal translocations. DNA Repair 5, 1093–1108. 10.1016/j.dnarep.2006.05.031 16857431

[B23] Frock R. L. Hu J. Meyers R. M. Ho Y. J. Kii E. Alt F. W. (2015). Genome-wide detection of DNA double-stranded breaks induced by engineered nucleases. Nat. Biotechnol. 33, 179–186. 10.1038/nbt.3101 25503383 PMC4320661

[B24] Ghamari A. van de Corput M. P. C. Thongjuea S. van Cappellen W. A. van IJcken W. van Haren J.. (2013). *In vivo* live imaging of RNA polymerase II transcription factories in primary cells. Genes Dev. 27, 767–777. 10.1101/gad.216200.113 23592796 PMC3639417

[B25] Ghezraoui H. Piganeau M. Renouf B. Renaud J. B. Sallmyr A. Ruis B.. (2014). Chromosomal translocations in human cells are generated by canonical nonhomologous end-joining. Mol. Cell 55, 829–842. 10.1016/j.molcel.2014.08.002 25201414 PMC4398060

[B26] Gittens W. H. Johnson D. J. Allison R. M. Cooper T. J. Thomas H. Neale M. J. (2019). A nucleotide resolution map of Top2-linked DNA breaks in the yeast and human genome. Nat. Commun. 10, 1–16. 10.1038/s41467-019-12802-5 31649282 PMC6813358

[B27] Gómez-González B. Aguilera A. (2019). Transcription-mediated replication hindrance: a major driver of genome instability. Genes Dev. 33, 1008–1026. 10.1101/gad.324517.119 31123061 PMC6672053

[B28] Gómez-Herreros F. Romero-Granados R. Zeng Z. Álvarez-Quilón A. Quintero C. Ju L.. (2013). TDP2–dependent non-homologous end-joining protects against topoisomerase ii–induced dna breaks and genome instability in cells and *in vivo*. PLoS Genet. 9:e1003226. 10.1371/journal.pgen.1003226 23505375 PMC3592926

[B29] Gómez-Herreros F. Schuurs-Hoeijmakers J. H. M. McCormack M. Greally M. T. Rulten S. Romero-Granados R.. (2014). TDP2 protects transcription from abortive topoisomerase activity and is required for normal neural function. Nat. Genet. 46, 516–521. 10.1038/ng.2929 24658003

[B30] Gómez-Herreros F. Zagnoli-Vieira G. Ntai I. Martínez-Macías M. I. Anderson R. M. Herrero-Ruíz A.. (2017). TDP2 suppresses chromosomal translocations induced by DNA topoisomerase II during gene transcription. Nat. Commun. 8:233. 10.1038/s41467-017-00307-y 28794467 PMC5550487

[B31] Gothe H. J. Bouwman B. A. M. Gusmao E. G. Piccinno R. Petrosino G. Sayols S.. (2019). Spatial chromosome folding and active transcription drive DNA fragility and formation of oncogenic MLL translocations. Mol. Cell 75, 267–283. 10.1016/j.molcel.2019.05.015 31202576

[B32] Guirouilh-Barbat J. Huck S. Bertrand P. Pirzio L. Desmaze C. Sabatier L.. (2004). Impact of the KU80 pathway on NHEJ-induced genome rearrangements in mammalian Cells. Mol. Cell 14, 611–623. 10.1016/j.molcel.2004.05.008 15175156

[B33] Haffner M. C. Aryee M. J. Toubaji A. Esopi D. M. Albadine R. Gurel B.. (2010). Androgen-induced TOP2B-mediated double-strand breaks and prostate cancer gene rearrangements. Nat. Genet. 42, 668–675. 10.1038/ng.613 20601956 PMC3157086

[B34] Hoa N. N. Shimizu T. Zhou Z. W. Wang Z. Q. Deshpande R. A. Paull T. T. (2016). Mre11 is essential for the removal of lethal topoisomerase 2 covalent cleavage complexes. Mol. Cell 64, 580–592. 10.1016/j.molcel.2016.10.011 27814490

[B35] Hu J. Meyers R. M. Dong J. Panchakshari R. A. Alt F. W. Frock R. L. (2016). Detecting DNA double-stranded breaks in mammalian genomes by linear amplification-mediated high-throughput genome-wide translocation sequencing. Nat. Protoc. 11, 853–871. 10.1038/nprot.2016.043 27031497 PMC4895203

[B36] Ju B. G. (2006). A topoisomerase II -mediated dsDNA break required for regulated transcription. Science 312, 1798–1802. 10.1126/science.1127196 16794079

[B37] Karanam K. Kafri R. Loewer A. Lahav G. (2012). Quantitative live cell imaging reveals a gradual shift between DNA repair mechanisms and a maximal use of HR in mid S phase. Mol. Cell 47, 320–329. 10.1016/j.molcel.2012.05.052 22841003 PMC3494418

[B38] Kent T. Chandramouly G. McDevitt S. M. Ozdemir A. Y. Pomerantz R. T. (2015). Mechanism of microhomology-mediated end-joining promoted by human DNA polymerase θ. Nat. Struct. Mol. Biol. 22, 230–237. 10.1038/nsmb.2961 25643323 PMC4351179

[B39] Kramara J. Osia B. Malkova A. (2018). Break-induced replication: the where, the why, and the how. Trends Genet. 34, 518–531. 10.1016/j.tig.2018.04.002 29735283 PMC6469874

[B40] Kumar-Sinha C. Tomlins S. A. Chinnaiyan A. M. (2008). Recurrent gene fusions in prostate cancer. Nat. Rev. Cancer 8, 497–511. 10.1038/nrc2402 18563191 PMC2711688

[B41] Kuo T. C. Schlissel M. S. (2009). Mechanisms controlling expression of the RAG locus during lymphocyte development. Curr. Opin. Immunol. 21, 173–178. 10.1016/j.coi.2009.03.008 19359154 PMC2676217

[B42] Kuzminov A. (2001). Single-strand interruptions in replicating chromosomes cause double-strand breaks. *Proc. Natl. Acad. Sci*. U.S.A. 98, 8241–8246. 10.1073/pnas.131009198 PMC37427 11459959

[B43] Lambert S. Saintigny Y. Delacote F. Amiot F. Chaput B. Lecomte M.. (1999). Analysis of intrachromosomal homologous recombination in mammalian cell, using tandem repeat sequences. Mutat. Res. Fund. Mol. Mecha. Mutag. 433, 159–168. 10.1016/s0921-8777(99)00004-x 10343649

[B44] Lensing S. V. Marsico G. Hänsel-Hertsch R. Lam E. Y. Tannahill D. Balasubramanian S. (2016). DSBCapture: in situ capture and sequencing of DNA breaks. Nat. Methods 13, 855–857. 10.1038/nmeth.3960 27525976 PMC5045719

[B45] Lieber M. R. (2010). The mechanism of double-strand DNA break repair by the nonhomologous DNA end-joining pathway. Annu. Rev. Biochem. 79, 181–211. 10.1146/annurev.biochem.052308.093131 20192759 PMC3079308

[B46] Lieber M. R. (2016). Mechanisms of human lymphoid chromosomal translocations. Nat. Rev. Cancer 16, 387–398. 10.1038/nrc.2016.40 27220482 PMC5336345

[B47] Lin C. Yang L. Tanasa B. Hutt K. Ju B. G. Ohgi K. A.. (2009). Nuclear receptor-induced chromosomal proximity and DNA breaks underlie specific translocations in cancer. Cell 139, 1069–1083. 10.1016/j.cell.2009.11.030 19962179 PMC2812435

[B48] Lin C. P. Ban Y. Lyu Y. L. Desai S. D. Liu L. F. (2008). A ubiquitin-proteasome pathway for the repair of topoisomerase I-DNA covalent complexes. J. Biol. Chem. 283, 21074–21083. 10.1074/jbc.M803493200 18515798 PMC2475699

[B49] Lindahl T. (1993). Instability and decay of the primary structure of DNA. Nature 362, 709–715. 10.1038/362709a0 8469282

[B50] Madabhushi R. (2018). The roles of DNA topoisomerase IIβ in transcription. Int. J. Mol. Sci. 19, 1917–1915. 10.3390/ijms19071917 29966298 PMC6073266

[B51] Madabhushi R. Gao F. Pfenning A. R. Pan L. Yamakawa S. Seo J.. (2015). Activity-induced DNA breaks govern the expression of neuronal early-response genes. Cell 161, 1592–1605. 10.1016/j.cell.2015.05.032 26052046 PMC4886855

[B52] Malkova A. Ira G. (2013). Break-induced replication: functions and molecular mechanism. Curr. Opin. Genet. Dev. 23, 271–279. 10.1016/j.gde.2013.05.007 23790415 PMC3915057

[B53] Mateos-Gomez P. A. Gong F. Nair N. Miller K. M. Lazzerini-Denchi E. Sfeir A. (2015). Mammalian polymerase θ promotes alternative NHEJ and suppresses recombination. Nature 518, 254–257. 10.1038/nature14157 25642960 PMC4718306

[B54] McVey M. Lee S. E. (2008). MMEJ repair of double-strand breaks (director's cut): deleted sequences and alternative endings. Trends Genet. 24, 529–538. 10.1016/j.tig.2008.08.007 18809224 PMC5303623

[B55] Mehta A. Haber J. E. (2014). Sources of DNA double-strand breaks and models of recombinational DNA repair. Cold Spring Harb. Perspect. Biol. 6:a016428. 10.1101/cshperspect.a016428 25104768 PMC4142968

[B56] Mertens F. Johansson B. Fioretos T. Mitelman F. (2015). The emerging complexity of gene fusions in cancer. Nat. Rev. Cancer 15, 371–381. 10.1038/nrc3947 25998716

[B57] Meyer C. Burmeister T. Gröger D. Tsaur G. Fechina L. Renneville A.. (2017). The MLL recombinome of acute leukemias in 2017. Leukemia 32, 273–284. 10.1038/leu.2017.213 28701730 PMC5808070

[B58] Meyer C. Schneider B. Reichel M. Angermueller S. Strehl S. Schnittger S.. (2005). Diagnostic tool for the identification of MLL rearrangements including unknown partner genes. Proc. Natl. Acad. Sci. U.S.A. 102, 449–454. 10.1073/pnas.0406994102 15626757 PMC544299

[B59] Mitelman F. Johansson B. Mertens F. (2007). The impact of translocations and gene fusions on cancer causation. Nat. Rev. Cancer 7, 233–245. 10.1038/nrc2091 17361217

[B60] Moynahan M. E. Jasin M. (1997). Loss of heterozygosity induced by a chromosomal double-strand break. Proc. Natl. Acad. Sci. U. S. A. 94, 8988–8993. 10.1073/pnas.94.17.8988 9256422 PMC22995

[B61] Moynahan M. E. Jasin M. (2010). Mitotic homologous recombination maintains genomic stability and suppresses tumorigenesis. Nat. Rev. Mol. Cell Biol. 11, 196–207. 10.1038/nrm2851 20177395 PMC3261768

[B62] Nitiss J. L. (2009a). DNA topoisomerase II and its growing repertoire of biological functions. Nat. Rev. Cancer 9, 327–337. 10.1038/nrc2608 19377505 PMC2730144

[B63] Nitiss J. L. (2009b). Targeting DNA topoisomerase II in cancer chemotherapy. Nat. Rev. Cancer 9, 338–350. 10.1038/nrc2607 19377506 PMC2748742

[B64] Osborne C. S. Chakalova L. Mitchell J. A. Horton A. Wood A. L. Bolland D. J.. (2007). Myc dynamically and preferentially relocates to a transcription factory occupied by Igh. PLoS Biol. 5:e192. 10.1371/journal.pbio.0050192 17622196 PMC1945077

[B65] Pfister S. X. Ahrabi S. Zalmas L. P. Sarkar S. Aymard F. Bachrati C. Z.. (2014). SETD2-dependent histone H3K36 trimethylation is required for homologous recombination repair and genome stability. CellReports 7, 2006–2018. 10.1016/j.celrep.2014.05.026 24931610 PMC4074340

[B66] Pommier Y. Sun Y. Huang S. Y. N. Nitiss J. L. (2016). Roles of eukaryotic topoisomerases in transcription, replication and genomic stability. Nat. Rev. Mol. Cell Biol. 17, 703–721. 10.1038/nrm.2016.111 27649880 PMC9248348

[B67] Ross J. A. (2000). Dietary flavonoids and the MLL gene: a pathway to infant leukemia? Proc. Natl. Acad. Sci. U.S.A. 97, 4411–4413. 10.1073/pnas.97.9.4411 10781030 PMC34309

[B68] Roukos V. Voss T. C. Schmidt C. K. Lee S. Wangsa D. Misteli T. (2013). Spatial dynamics of chromosome translocations in living cells. Science 341, 660–664. 10.1126/science.1237150 23929981 PMC6324928

[B69] Rulten S. L. Caldecott K. W. (2013). DNA strand break repair and neurodegeneration. DNA Repair 12, 558–567. 10.1016/j.dnarep.2013.04.008 23712058

[B70] Sakofsky C. J. Malkova A. (2017). Break induced replication in eukaryotes: mechanisms, functions, and consequences. Crit. Rev. Biochem. Mol. Biol. 52, 395–413. 10.1080/10409238.2017.1314444 28427283 PMC6763318

[B71] Sasanuma H. Tsuda M. Morimoto S. Saha L. K. Rahman M. M. Kiyooka Y.. (2018). BRCA1 ensures genome integrity by eliminating estrogen-induced pathological topoisomerase II–DNA complexes. Proc. Natl. Acad. Sci. U.S.A. 115, E10642–E10651. 10.1073/pnas.1803177115 30352856 PMC6233096

[B72] Schellenberg M. J. Lieberman J. A. Herrero-Ruíz A. Butler L. R. Williams J. G. Muñoz-Cabello A. M.. (2017). ZATT (ZNF451)–mediated resolution of topoisomerase 2 DNA-protein cross-links. Science 357, 1412–1416. 10.1126/science.aam6468 28912134 PMC5623066

[B73] Schwer B. Wei P. C. Chang A. N. Kao J. Du Z. Meyers R. M.. (2016). Transcription-associated processes cause DNA double-strand breaks and translocations in neural stem/progenitor cells. Proc. Natl. Acad. Sci. U.S.A. 113, 2258–2263. 10.1073/pnas.1525564113 26873106 PMC4776469

[B74] Sfeir A. Symington L. S. (2015). Microhomology-mediated end joining: a back-up survival mechanism or dedicated pathway? Trends Biochem. Sci. 40, 701–714. 10.1016/j.tibs.2015.08.006 26439531 PMC4638128

[B75] So C. C. Martin A. (2019). DSB structure impacts DNA recombination leading to class switching and chromosomal translocations in human B cells. PLoS Genet. 15:e1008101. 10.1371/journal.pgen.1008101 30946744 PMC6467426

[B76] Stark J. M. Jasin M. (2003). Extensive loss of heterozygosity is suppressed during homologous repair of chromosomal breaks. Mol. Cell. Biol. 23, 733–743. 10.1128/MCB.23.2.733-743.2003 12509470 PMC151548

[B77] Symington L. S. (2016). Mechanism and regulation of DNA end resection in eukaryotes. Crit. Rev. Biochem. Mol. Biol. 51, 195–212. 10.3109/10409238.2016.1172552 27098756 PMC4957645

[B78] Tsai S. Q. Zheng Z. Nguyen N. T. Liebers M. Topkar V. V. Thapar V.. (2014). GUIDE-seq enables genome-wide profiling of off-target cleavage by CRISPR-Cas nucleases. Nat. Biotechnol. 33, 187–197. 10.1038/nbt.3117 25513782 PMC4320685

[B79] Uusküla-Reimand L. Hou H. Samavarchi-Tehrani P. Rudan M. V. Liang M. Medina-Rivera A. (2016). Topoisomerase II beta interacts with cohesin and CTCF at topological domain borders. Genome Biol. 1:182 10.1186/s13059-016-1043-8 PMC5006368 27582050

[B80] Vos S. M. Tretter E. M. Schmidt B. H. Berger J. M. (2011). All tangled up: how cells direct, manage and exploit topoisomerase function. Nat. Rev. Mol. Cell Biol. 12, 827–841. 10.1038/nrm3228 22108601 PMC4351964

[B81] Wei P. C. Chang A. N. Kao J. Du Z. Meyers R. M. Alt F. W.. (2016). Long neural genes harbor recurrent DNA break clusters in neural stem/progenitor cells. Cell 164, 644–655. 10.1016/j.cell.2015.12.039 26871630 PMC4752721

[B82] Whitmarsh R. J. Saginario C. Zhuo Y. Hilgenfeld E. Rappaport E. F. Megonigal M. D.. (2003). Reciprocal DNA topoisomerase II cleavage events at 5“-TATTA-3” sequences in MLL and AF-9 create homologous single-stranded overhangs that anneal to form der(11) and der(9) genomic breakpoint junctions in treatment-related AML without further processing. Oncogene 22, 8448–8459. 10.1038/sj.onc.1207052 14627986

[B83] Wright R. L. Vaughan A. T. M. (2014). A systematic description of MLL fusion gene formation. Crit. Rev. Oncol. Hematol. 91, 283–291. 10.1016/j.critrevonc.2014.03.004 24787275

[B84] Wright W. D. Shah S. S. Heyer W.-D. (2018). Homologous recombination and the repair of DNA double-strand breaks. J. Biol. Chem. 293, 10524–10535. 10.1074/jbc.TM118.000372 29599286 PMC6036207

[B85] Xu Z. Zan H. Pone E. J. Mai T. Casali P. (2012). Immunoglobulin class-switch DNA recombination: induction, targeting and beyond. Nat. Rev. Immunol. 12, 517–531. 10.1038/nri3216 22728528 PMC3545482

[B86] Yan C. T. Boboila C. Souza E. K. Franco S. Hickernell T. R. Murphy M.. (2007). IgH class switching and translocations use a robust non-classical end-joining pathway. Nature 449, 478–482. 10.1038/nature06020 17713479

[B87] Yan W. X. Mirzazadeh R. Garnerone S. Scott D. Schneider M. W. Kallas T.. (2017). BLISS is a versatile and quantitative method for genome-wide profiling of DNA double-strand breaks. Nat. Commun. 8, 1–9. 10.1038/ncomms15058 28497783 PMC5437291

[B88] Yu X. Davenport J. W. Urtishak K. A. Carillo M. L. Gosai S. J. Kolaris C. P.. (2017). Genome-wide TOP2A DNA cleavage is biased toward translocated and highly transcribed loci. Genome Res. 27, 1238–1249. 10.1101/gr.211615.116 28385713 PMC5495075

[B89] Zagnoli-Vieira G. Bruni F. Thompson K. He L. Walker S. de Brouwer A. P. M.. (2018). Confirming TDP2 mutation in spinocerebellar ataxia autosomal recessive 23 (SCAR23). Neurol. Genet. 4:e262. 10.1212/NXG.0000000000000262 30109272 PMC6089694

[B90] Zahn K. E. Averill A. M. Aller P. Wood R. D. Doublié S. (2015). Human DNA polymerase θ grasps the primer terminus to mediate DNA repair. Nat. Struct. Mol. Biol. 22, 304–311. 10.1038/nsmb.2993 25775267 PMC4385486

[B91] Zeng Z. Cortes-Ledesma F. El Khamisy S. F. Caldecott K. W. (2010). TDP2/TTRAP Is the major 5′-tyrosyl DNA phosphodiesterase activity in vertebrate cells and is critical for cellular resistance to topoisomerase II-induced DNA damage. J. Biol. Chem. 286, 403–409. 10.1074/jbc.M110.181016 21030584 PMC3012998

[B92] Zhang A. Lyu Y. L. Lin C. P. Zhou N. Azarova A. M. Wood L. M.. (2006). A protease pathway for the repair of topoisomerase II-DNA covalent complexes. J. Biol. Chem. 281, 35997–36003. 10.1074/jbc.M604149200 16973621

[B93] Zhang Y. Jasin M. (2010). An essential role for CtIP in chromosomal translocation formation through an alternative end-joining pathway. Nat. Struct. Mol. Biol. 18, 80–84. 10.1038/nsmb.1940 21131978 PMC3261752

[B94] Zhang Y. McCord R. P. Ho Y. J. Lajoie B. R. Hildebrand D. G. Simon A. C.. (2012). Spatial organization of the mouse genome and its role in recurrent chromosomal translocations. Cell 148, 908–921. 10.1016/j.cell.2012.02.002 22341456 PMC3320767

